# The Role of Innate Immune System in the Human Amniotic Membrane and Human Amniotic Fluid in Protection Against Intra-Amniotic Infections and Inflammation

**DOI:** 10.3389/fimmu.2021.735324

**Published:** 2021-10-21

**Authors:** Tina Šket, Taja Železnik Ramuta, Marjanca Starčič Erjavec, Mateja Erdani Kreft

**Affiliations:** ^1^ Department of Synthetic Biology and Immunology, National Institute of Chemistry, Ljubljana, Slovenia; ^2^ Institute of Cell Biology, Faculty of Medicine, University of Ljubljana, Ljubljana, Slovenia; ^3^ Department of Biology, Biotechnical Faculty, University of Ljubljana, Ljubljana, Slovenia

**Keywords:** human amniotic membrane, placenta, intrauterine infection, innate immune system, antimicrobial activity, bacteria, preterm birth

## Abstract

Intra-amniotic infection and inflammation (IAI) affect fetal development and are highly associated with preterm labor and premature rupture of membranes, which often lead to adverse neonatal outcomes. Human amniotic membrane (hAM), the inner part of the amnio-chorionic membrane, protects the embryo/fetus from environmental dangers, including microbial infection. However, weakened amnio-chorionic membrane may be breached or pathogens may enter through a different route, leading to IAI. The hAM and human amniotic fluid (hAF) respond by activation of all components of the innate immune system. This includes changes in 1) hAM structure, 2) presence of immune cells, 3) pattern recognition receptors, 4) cytokines, 5) antimicrobial peptides, 6) lipid derivatives, and 7) complement system. Herein we provide a comprehensive and integrative review of the current understanding of the innate immune response in the hAM and hAF, which will aid in design of novel studies that may lead to breakthroughs in how we perceive the IAI.

## Introduction

Amniotic membrane (AM) serves as a wall of an embryo/fetal annex, and is the innermost component of the fetal membrane (i.e. amnio-chorionic membrane) that envelops the amniotic fluid (AF) with the developing embryo/fetus (1). The evolutionary need for AM is thought to emerge concurrently with the transition of animals from water to land (2). The membrane of the so-called amniotic egg provided protection from dryer environment, while allowing the necessary gas exchange (3). These egg-laying animals are ancestors to today’s clade Amniotes, which comprises of reptiles, birds and mammals. Though there are significant differences between the AM and AF of these animals, its main functions remain the same: to provide nourishment, homeostatic environment and protection from physical, chemical and biological stress to the fetus (4–7).

## Human Amniotic Membrane

Human AM (hAM), originates from the embryo and forms a multilayer structure that is between 0.02 and 0.5 mm thick (8–10). Human AF (hAF) is in direct contact with a monolayer of hAM epithelial cells (hAEC), which are attached to the basement membrane that borders on the compact layer of hAM. Next is the fibrous layer with hAM mesenchymal stromal cells (hAMSC), and finally the spongy layer of hAM, which adheres the human chorionic membrane (hCM) composed of reticular layer, basement membrane and chorion trophoblast cells (9, 10).

At term a subpopulation of hAEC expresses embryonic stem cell surface markers, such as tumor rejection antigens 1-60 (TRA1-60) and TRA1-81 (11–13), stage-specific embryonic antigens 3 and 4 (SSEA-3, SSEA-4) (11, 13, 14), and also some transcription factors characteristic for stem cells, such as OCT-4, NANOG, SOX-2 and SOX-3 (11, 12, 15, 16). Similarly, a subpopulation of hAMSC also expresses some of the pluripotency markers, namely TRA-1-61, TRA1-81 (17), OCT-3 (18), OCT-4 (17–19), SSEA-3, SSEA-4 (17, 20), SOX-2 and NANOG (19, 20). Moreover, hAEC and hAMSC are capable of differentiation into all three germ layers, namely ectoderm, mesoderm and endoderm (15, 17, 21).

Besides the nourishment and homeostatic functions, the cells of the hAM provide an extensive immune defense against the potential pathogens. hAM cells and cells involved in the immune response secrete a vast array of protective molecules including lipids, peptides, and proteins that comprise up to 25% of all identified proteins found in the hAF (22). Overall, a large number of identified molecules and complexes related to immune defense (22) appear redundant, but offer a possibly synergistic protection against various pathogens (22, 23).

At first, hAEC and hAMSC have been considered to be immune privileged, but it has been shown that they are able to elicit immune responses under certain conditions (24–26). The anti-inflammatory activity of hAM-derived cells has been well documented. hAM-derived cells and their conditioned medium block differentiation and maturation of monocytes into dendritic cells or inflammatory M1 macrophages (27–32). Moreover, when the M1 macrophages are cultured with hAMSC or their conditioned medium, their expression of co-stimulatory proteins CD80, CD86 and CD40, decreases. The hAMSC and their conditioned medium have been shown to shift differentiation of monocytes (under M1 differentiation conditions) towards the anti-inflammatory M2 phenotype and they also reduce the pro-inflammatory cytokine secretion interleukin (IL)-1α, IL-1β, IL-12, IL-8, tumor necrosis factor alpha (TNFα), macrophage inflammatory protein (MIP)1α, MIP1β, monokine induced by gamma interferon, regulated on activation, normal T expressed and secreted (RANTES), interferon-gamma inducible protein (IP)-10) by the M2 macrophages, while increasing the secretion of the anti-inflammatory cytokine IL-10 (32).

The hAEC inhibit neutrophil (33) and macrophage migration by secretion of the migration inhibitor factor (MIF) (34), while the hAMSC reduce neutrophil migration (35). The hAEC and hAMSC have also been shown to inhibit NK cell cytotoxicity and reduce IFN-gamma expression in a dose-dependent manner (36).

The versatile nature of hAM-derived cells is well demonstrated by their ability to induce immunosuppression as well as immunostimulation. Namely, when hAM-derived cells are co-cultured with unstimulated allogenic peripheral blood mononuclear cells (PBMCs) at low concentrations, they have been shown to stimulate PBMCs proliferation (25, 30, 37). Similarly, the co-culture of T lymphocytes with low concentrations of hAM-derived cells led to induction of proliferation in T lymphocytes (25, 31). Therefore, the hAM may function as a sensor and regulator of the inflammatory response to infections and/or inflammatory stimuli (38). Interestingly, there is a great shortage of studies (especially in the *in vivo* setting) that would investigate the immunomodulatory activity of hAM in the context of IAI. The studies investigating the expression and production of immunomodulatory molecules by hAM cells in IAI are described in the Chapter 3.

It is thought that at term, amnio-chorionic membrane weakening that occurs due to irreversible epithelial-to-mesenchymal transition (EMT), as well as the initiation of labor are inflammation dependent, and can be triggered by physical stretching of the membrane and an increased oxidative stress (39, 40). The resulting cellular stress produces Damage-Associated Molecular Patterns (DAMPs) and senescence-associated secretory phenotypes (SASPs), in the case of infection also Pathogen-Associated Molecular Patterns (PAMPs), which contribute to the necessary inflammation through downstream signalling cascades (40–42). Moreover, hAM was shown to be crucial in the production of prostaglandins, which induce cervical ripening and uterine contractions (43, 44).

## Intra-Amniotic Infection And Inflammation

Occasional infections of the amnio-chorionic membranes or hAF do occur and may harm the fetus or cause preterm labor and other complications (45, 46). Infections are present in 15-35% of all pregnancies that result in preterm birth (47, 48). As infection and inflammation are associated processes, it is sometimes difficult to distinguish between them, especially in cases when only limited analysis or sampling is available. Therefore, the term chorioamnionitis includes inflammation of intrauterine structures amnion and chorion, their infection, or both; in this paper we refer to this as intra-amniotic infection/inflammation (IAI) (49).

IAIs are most commonly caused by pathogenic bacteria and fungi, such as *Mycoplasma* sp., *Ureaplasma* sp., *Bacteroides* sp., group B *Streptococcus* sp., *Escherichia coli*, *Enterococcus faecalis*, *Klebsiella pneumoniae, Fusobacterium nucleatum*, *Gardnerella vaginalis* and *Candida albicans* (50, 51) and viruses, such as cytomegalovirus, herpes simplex virus types 1 and 2 and Zika virus (52, 53). SARS-Cov-2 that caused the current pandemic can in rare cases infect the fetus through a transplacental transmission (54, 55). Interestingly, IAI is often caused by multiple pathogens, since over 65% of all positive hAF cultures involve two or more microorganisms (56). While the identified pathogens are diverse, they are mainly typical genital pathogens, largely from the phylum Firmicutes, order Mycoplasmatales (57).

The question whether the placenta has its own microbiome, or if the presence of bacteria always indicates potential pathogens still remains open. A study performed by Aagaard et al. (58) demonstrated the presence of microbiome in human placenta, which was low in abundance but metabolically rich (58). Moreover, others have demonstrated the presence of microbiota in the human umbilical cord blood and hAF (59, 60). On the other hand, these findings were refuted by de Goffau et al. (61) who investigated the presence of bacterial DNA in the human placenta and demonstrated that the human placenta has no microbiome but can contain potential pathogens (61). Furthermore, not only was there no evidence for the presence of bacteria in the majority of samples, but all signs were related to the acquisition of bacteria during labor and delivery or to contamination of laboratory reagents with bacterial DNA (61–63). The contradicting observations of these studies reflect the difficulty of studying placental microbiome, since the placenta is a relatively inaccessible tissue during pregnancy, and after birth it is easily contaminated. Furthermore, the microbiome, if there is one, is limited in the amount of microbes, and therefore challenging to detect. This is especially true for less sensitive methods, such as classical cultivation techniques, whereas DNA amplification techniques are more sensitive but are also more prone to contamination and present an indirect evidence of microbes. In the future, further investigations will need to be done to answer the questions of presence and extent of placental microbiome.

Multiple routes of invasion have been proposed for IAI. Namely, pathogens can enter the amniotic cavity by 1) ascending migration from the lower genital tract through the cervix (most common route of infection), 2) haematogenous dissemination from distant sites (e.g. the intestine or oral cavity) through the placenta, 3) retrograde seeding from the peritoneal cavity through the fallopian tubes or 4) iatrogenic introduction during the invasive procedures (57, 64, 65). Interestingly, a study by Kim et al. (66) indicates that the microbial invasion of the amniotic cavity does not follow widespread infection of amnio-chorionic membrane, but precedes it. The authors proposed that the microbial invasion of the amniotic cavity starts by intra-amniotic bacterial invasion through a discrete region of the amnio-chorionic membrane, is followed by intra-amniotic proliferation and therefore, bacterial invasion of amnio-chorionic membrane primarily extends from the hAF (66). The pathogens are recognized by the pattern recognition receptors (PRRs), a component of the innate immune system, which activates the synthesis of cytokines, chemoattractant cytokines (chemokines), prostaglandins, proteases, antimicrobial peptides, and other mechanisms of the innate immune system, some of which are mediated by neutrophils, monocytes and lymphocytes (51, 67, 68). This immune cascade can lead to premature contractions, cervical changes and premature delivery. Furthermore, some bacteria are even capable of producing enzymes that degrade amnio-chorionic membrane or inducing synthesis and release of molecules that stimulate uterine contractions and lead to preterm labor (e.g. prostaglandins) (57, 69). Importantly, various pathogens and the inflammatory response that follows the infection, affect the fetal development (especially the developing fetal lung and brain), and may also impact the long-term health of the infant (70). IAI can affect different neurological developmental processes, inducing neuroinflammation, cerebral palsy, and periventricular white matter injury in the fetus, and even neuropsychiatric diseases have been linked to IAI (71–76). Chronically damaged lungs in the form of bronchopulmonary dysplasia are also highly associated with IAI (77–79).

The aim of this review is therefore to encompass the variability and importance of the innate immune response of the hAM and hAF implicated in the IAI. To provide a thorough overview of the literature, we collected an unbiased set of articles covering all parts of the hAM and hAF innate immune response related to IAI during pregnancy and in labor. The studies were screened and selected from PubMed database using key words “intraamniotic”, “infection”, “amnion”, and various components of the innate immune system. Studies, which were investigating immune response in tissues other than hAM or hAF were excluded. Several *in vitro* studies that used hAM or its derivatives are also considered, and an overview of animal models for IAI is presented in a recent paper by Cappelletti et al. (80).

## Innate Immune System Defense Against Pathogens in the hAF and hAM

**Figure 1:**
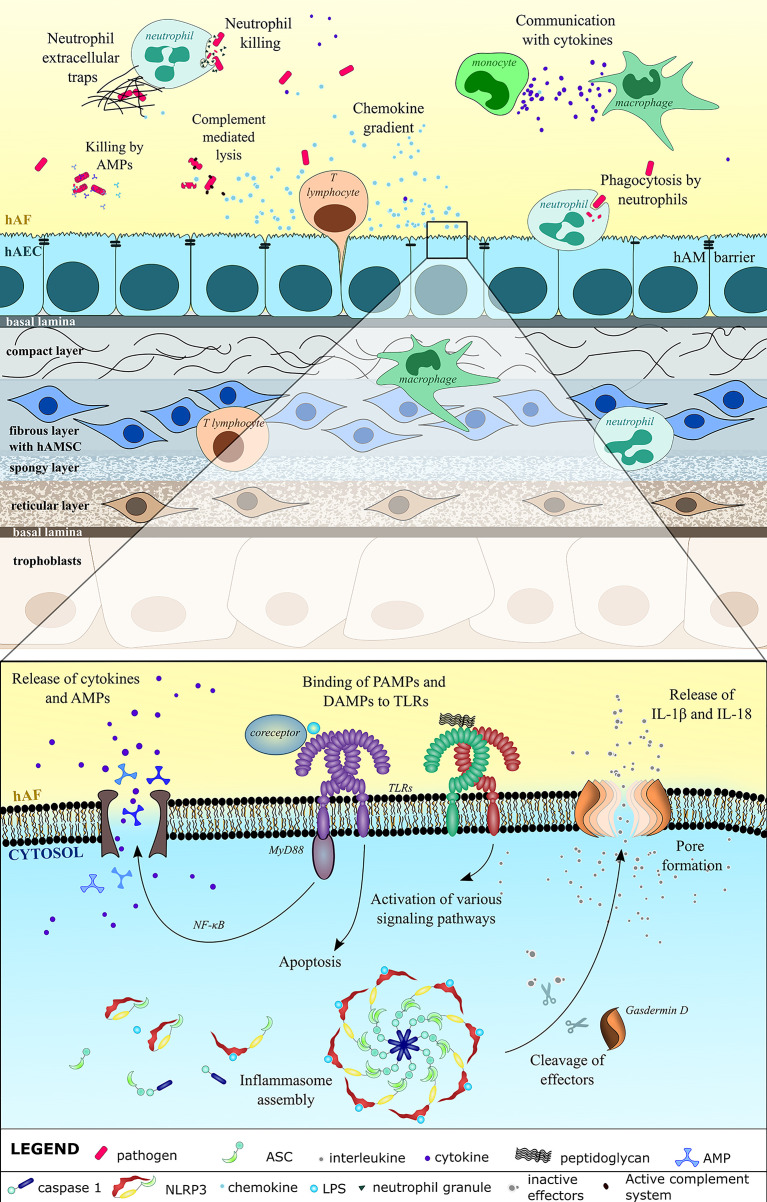
Innate immune system defense against pathogens in the hAF and hAM is comprehensive. hAM integrity prevents the entry of pathogens. Breaching of hAM causes upregulated expression and release of cytokines, including chemokines, which engage immune cells neutrophils, macrophages, monocytes and T lymphocytes. Immune cells regulate the inflammatory process and directly kill the microbes through phagocytosis, formation of neutrophil extracellular traps, and direct killing with granules. Using the pattern recognition receptors (PRRs), the hAEC recognize the pathogen-associated molecular patterns (PAMPs) (LPS, bacterial glycoprotein) and damage-associated molecular patterns (DAMPs) that are a result of the microbial infection. Most notably, these include toll-like receptors (TLRs) on cell membrane and the nucleotide-binding oligomerization domain (NOD)- Leucin Rich Repeats (LRR)-containing receptors (NLRs) in the cytosol, both of which induce various downstream signaling that culminates in the release and maturation of inflammatory cytokines, regulated cell death and secretion of antimicrobial peptides (AMPs). AMPs and activated complement system directly damage pathogens, mainly by targeting their cell wall and by causing lysis.

The immune system of the fetus and (preterm) neonate is immature and requires stimulation to fully develop. Moreover, the fetal adaptive immune responses are downregulated, which may enhance their vulnerability to infection but at the same time this has a protective role against collateral inflammatory damage and is related to maintaining the intricate immune balance between the mother and the fetus (62, 81). Therefore, fetuses and neonates largely rely on their innate immune system and additional protection is provided by various components of the placenta, namely by the amnio-chorionic membrane and decidua and also by the hAF (62, 80, 82, 83). The studies that explored the involvement of the innate immune system in the hAM and hAF in relation to the aforementioned IAIs are summed up in the following subsections, and in **Figure 1** and **Table 1**. The involvement of other important tissues in immune response in IAI is not covered in this review.

**Table 1:** Response of various components of the innate immune system to the intra-amniotic infection/inflammation.

Component of the innate immune system		Function	Response to the intra-amniotic infection
**hAM as a physical barrier**		Prevention of microbial entry	The presence of inflammatory mediators leads to the loss of tight junctions between the hAEC and an increased level of apoptosis, senescence and necrosis (84–92).
			The EMT transitions of hAEC can weaken the hAM and contribute to the onset of parturition (90–94).
			The endogenous host response to microbial infections includes secretion of extracellular matrix degrading enzymes matrix metalloproteinases (93, 95–106).
			The invading microbes may produce their own extracellular matrix-degrading enzymes (107).
			Loss of hAM’s integrity increases chances of PROM (90–92, 108–111).
**Immune cells**	Neutrophils	Production of reactive oxygen species that are cytotoxic to microbes, phagocytosis, production of antimicrobial peptides and cytokines	The hAF neutrophils can be predominantly of the fetal or maternal origin or a mixture of both (112, 113).
			The hAF neutrophils can invade the amniotic cavity, therefore, the fetus and the mother participate in the host defense mechanisms against intra-amniotic infection (113–118).
			The hAF neutrophils phagocytose bacteria and form neutrophil extracellular traps (113–115, 118).
	Monocytes/Macrophages	Production of nitric oxide that is cytotoxic to microbes, phagocytosis, production of cytokines	Monocytes/macrophages can be predominantly of the fetal or maternal origin or a mixture of both (119).
			Monocytes/macrophages in the amniotic cavity primarily act through the release of pro-inflammatory cytokines (120, 121).
**Pattern recognition receptors (PRRs)**	Toll-like receptors (TLRs)	Recognition of conserved features of microbes and downstream signaling	PRRs induce inflammation through the activation of several inflammatory pathways (122–131).
	Nucleotide-binding oligomerization domain (NOD)- Leucine-Rich Repeats (LRR)-containing receptors (NLRs)		Intra-amniotic infection leads to an increase of the transcriptional level of NLRP1, NLRP3, NLRC4, NOD2 (118).
	Retinoic acid-inducible gene 1 (RIG-1)-like receptors (RLRs)		Activation of the NLRP3 inflammasome promotes preterm birth (118, 132–138).
	C-type lectin receptors (CLRs)		TLR1-10 are expressed by the hAEC (122, 139).
**Cytokines**		Signaling molecules that mediate and regulate immunity, inflammation and hematopoiesis	Presence of IL-6, IL-1β, IL-10, IL-18, TNFα, macrophage migration inhibitory factor, nicotinamide phosphoribosyltransferase, TGFβ, granulocyte-macrophage colony-stimulating factor, high-mobility group protein 1, IL-10 and IL-6 in the hAF with IAI or preterm labor conditions (118, 121, 128, 129, 140–153).
			An elevated level of cytokines IL-1β, IL-6, TNFα, IFNγ, EGF, MIP3α in patients with PROM and intra-amniotic infection (154).
			The intra-amniotic infection leads to an increased level of cytokines and chemokines IL-1b, IL-6, IL-8, TNFα, IFNγ, EGF, MIP3α, MIP1α, Eotaxin, IL-16, IL-8, monocyte chemoattractant protein-1, chemokine (C-X-C motif) ligands (CXCLs)-1, -3, -4, -5, -6, -10, -11 and L-selectin (128, 146, 147, 154–159).
**Antimicrobial peptides**	α- and β-defensins	AMPs damage and kill bacteria mainly by disrupting their membrane	AMPs are expressed by the hAM cells and are present in the hAF (23, 160–176).
	SLPI		Intra-amniotic infection leads to increased levels of AMPs (23, 100–102, 150, 152, 162, 166, 168–174, 177–183).
	Elafin		
	Calgranulin/Calprotectin		
	Lactoferrin		
	Lipocalin 2		
	Cathelicidin		
**Lipids and derivatives**	Prostaglandins	Bioactive molecules that mediate human parturition	Intra-amniotic infection leads to an increased prostaglandin-prostamide ratio (122, 139, 184–197).
	5-lipoxygenase pathway molecules		Intra-amniotic infection causes an increase of molecules of 5-lipoxygenase pathway (191, 198).
**Complement system**	Complement molecules C3a, Bb	Complement activation lyses pathogens and regulates innate and adaptive immune response	C3a and Bb are increased in the hAF of women with intra-amniotic infection (199, 200).
	Inhibitor CD-59		
**Other molecules and pathways**	Glycolysis	Various - consequence of infection/inflammation or unknown	Upon intra-amniotic infection or inflammation numerous molecules show changed concentration in the hAF (102, 174, 178, 201–211).
	Gluconeogenesis		
	Iron homeostasis		
	Immune cell products		
	Other		

### AM as a Physical Barrier

Amnio-chorionic membrane, including hAM, present a barrier for the potential intra-amniotic pathogens. Their loss of integrity, inflammation, and inflammation-related oxidative stress can increase the chance of preterm rupture of membranes (PROM) or directly permit the entry of pathogens to the hAF, which sequentially weakens the membrane further (212, 213). hAM integrity can be modified by changes in the extracellular matrix modified by a pro-inflammatory environment. For example, activin A, which is involved in connective tissue remodeling, is increased in IAI, and its stimulation by LPS or TNFα suppresses type I and type III collagen mRNA levels in hAMSC (84–86). Additionally, the amount of extracellular matrix glycoprotein tenascin X was changed with IAI (87) and an *in vitro* model hAMSC suppressed expression of collagen types I and III with activin A stimulation (85). Matrix metalloproteinases (MMPs) are important regulators of inflammation with their role extending beyond the degradation of extracellular matrix (214). The relationship between MMPs and fetal membrane weakening and preterm labor is reviewed by Vadillo-Ortega et al. (95). Studies investigating association of IAI and PROM with MMP concentration discovered the upregulation of MMP-7, MMP-8 and MMP-9, with inconsistent conclusions for MMP-2 (93, 96–103). The discrepancies for MMP-2 are the result of different experimental settings; the studies by Fortunato et al. (96) and Myntti et al. (102) have seen an increase in total levels of MMP-2 and in catalitically active levels of MMP-2 (tissue inhibitor of metalloproteinases (TIMP)-free MMP2) in the hAF samples, while Flores-Herrera et al. (99) used an *in vitro* model of infected amnio-chorionic membrane and did not observe an increase in the secretion of proMMP-2. MMP inhibitor TIMP1 was increased in preterm PROM and in IAI, while TIMP2 was decreased in preterm PROM (96, 99, 102). Similarly, a higher ratio of MMP : TIMP was observed also in the placenta and in maternal serum in cases of preterm birth (104, 105). Interestingly, the altered levels of MMPs and their inhibitors in the plasma of preterm infants appear to have a role in the development of bronchopulmonary dysplasia and intraventricular hemorrhage (106). Overall, there are still very few results concerning MMPs, therefore, definitive conclusions are still missing. Fetal membrane weakening can also be caused by prothrombin, which was shown to be upregulated in hAM when stimulated with *Ureaplasma parvum* in an *in vitro* study (108–110). Moreover, the disruption of tight junctions between AECs was observed and characterized by the loss of claudins 3 and 4 in mice, where IAI was induced with lipopolysaccharide (LPS) administration (88). In the same setting, apoptosis of the AECs was also observed. Studies of hAM samples, obtained at term, show high levels of endocytosis and autophagy, thought to be induced by lack of nutrients and possibly hormonal changes during the end of pregnancy (215). Autophagic death and apoptosis of hAECs are elevated at ruptured sites of the hAM, signifying their involvement in the weakening and rupture of hAM at term (216). Nevertheless, more studies are needed to decipher the role of different intrinsic cell deaths of hAECs in IAI. Infection of hAM cell line by *Listeria monocytogenes*, a rare but very dangerous pathogen in IAI, results in cell necrosis after bacteria have successfully multiplied, suggesting yet another way of hAM weakening (89).

**Figure 2:**
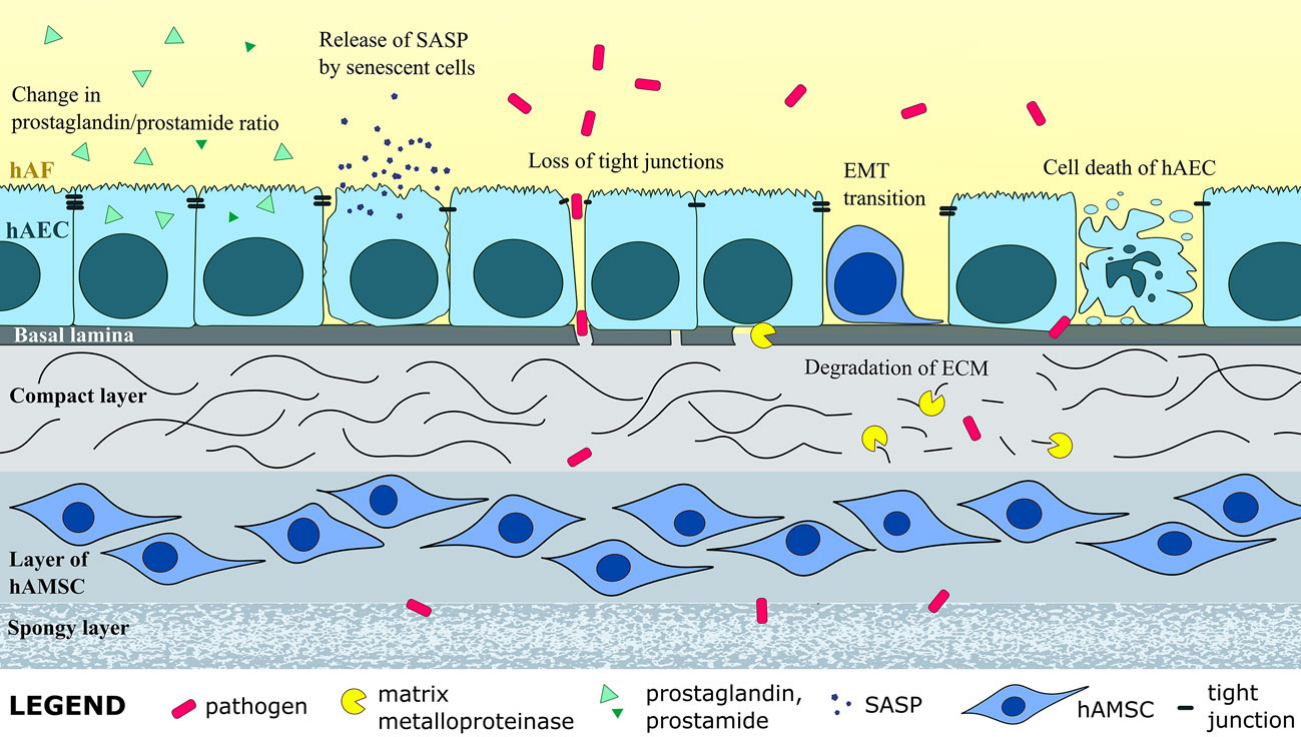
Intra-amniotic infection and inflammation induce structural changes in the hAM, such as enzymatic degradation of extracellular matrix (ECM), EMT of hAEC, senescence, apoptosis and necrotic death of hAEC, and loss of tight junctions between hAEC, all of which weakens the hAM and can cause PROM. Change in levels of numerous molecules, as well as prostaglandin-prostamide ratio, can induce labor and lead to preterm labor.

Infection, inflammation, and related oxidative stress promote cell senescence of hAEC, and have been observed in hAM of premature PROM (90–92). Throughout pregnancy, EMT and the reverse mesenchymal-to-epithelial transition (MET) contribute to healing of small hAM ruptures and the maintenance of hAM integrity during the fetal growth (217). The process is partially regulated by oxidative stress and transforming growth factor β, which promote EMT, and by progesterone, which promotes MET (39, 218, 219). However, at term the homeostasis of hAM remodeling *via* cellular transitions is disrupted by irreversible EMT of hAECs, which is necessary for weakening of the membrane and parturition (92). Furthermore, *in vitro* studies with hAECs were shown to undergo EMT when stimulated with TNFα (93, 94). Therefore, an imbalance in EMT and MET may occur due to IAI and related oxidative stress, and may promote premature PROM. Altogether, the considerable changes to the extracellular matrix and hAM cells during IAI can weaken the hAM, promote preterm onset of labor, and allow further complications for the pregnancy (**Figure 2**).

### Immune Cells

In the hAF of women with IAI the total leukocyte count is up to 100-fold increased (68, 120, 140, 155, 220, 221). The leukocytes are also elevated in preterm PROM, especially in extremely early gestation (103). There is a general increase in T lymphocyte numbers associated with hAF inflammation, which is largely due to T helper cells, although NK and cytotoxic T lymphocytes have been implicated as well (68, 120, 221, 222). Importantly, the number of phagocytic monocytes/macrophages and neutrophils is drastically higher in the hAF of IAI cases (68, 114, 120, 221–223). Neutrophils found in the hAF during IAI, can mostly originate from the mother, or fetus, or both (112, 113), and have been shown to phagocytose bacteria and form neutrophil extracellular traps (NETs) (114, 115). NETs are actively formed by a process called NETosis, during which the chromatin and antimicrobial proteins are released into neutrophil vicinity, thereby entrapping, disarming, and killing the pathogens (224). Unsurprisingly, the immune cells are also present in amnio-chorionic membrane, and the presence (and abundance) of neutrophils or lymphocytes defines the stage of acute or chronic chorioamnionitis, respectively (116, 117). Neutrophils and monocytes/macrophages are most abundant in hAM in acute chorioamnionitis and natural killer cells can also be found, while CD3+ and CD8+ lymphocytes are present in moderate amounts in chronic chorioamnionitis with smaller number of CD4+ (helper T) cells (115–117, 141, 225–227). As is explained in the reviews by Cappelletti et al. (80) and Hoo et al. (227), during normal pregnancy, the decidual-placental interface is rich with immune cells, which become activated in IAI and migrate towards amnio-chorionic membrane (80). The immune cells involved in the inflammation are of maternal and fetal origin, and must maintain a delicate balance of eliminating an infection, while preventing the damage to the fetus. The role of both populations, maternal and fetal, should be further studied. A recent paper by Gomez-Lopez (228) suggests that the activation of fetal T lymphocytes in the hAF may be enough to cause preterm birth in some cases (228). The presence and activation of immune cells, however, is the result of a complex regulatory network that begins with the identification of pathogens or their components.

### Pattern Recognition Receptors

Pattern recognition receptors are proteins that have evolved to recognize conserved features of microbes PAMPs and molecules released by damaged cells DAMPs (229, 230). PRRs can be divided into four major groups, namely 1) Toll-like receptors (TLRs), 2) the nucleotide-binding oligomerization domain (NOD)- Leucin Rich Repeats (LRR)-containing receptors (NLRs), 3) the retinoic acid-inducible gene 1 (RIG-1)-like receptors (RLRs) and 4) the C-type lectin receptors (CLRs) (229, 231). The engagement of PRRs on the cells of the innate immune system induces co-stimulatory signals for the cells of the adaptive immune system (229, 232, 233). DAMPs and PAMPs, which trigger PRRs, can indirectly cause tissue remodeling and fetal membrane weakening, though the regulation of these processes in the context of IAI has not been elucidated yet (40). Furthermore, hAECs release exosomes packed with inflammatory mediators, such as DAMPs, PAMPs and SASP, which induce inflammation in uterine tissue that promotes (preterm) labor (213, 234–236).

TLRs1-10 are expressed in hAEC and hAMSC at transcriptional level under normal (non-IAI) conditions at term (122, 237, 238). Specifically, TLR1, TLR2, TLR4, TLR5, TLR7, TLR8, TLR9, and heterodimer TLR6/2 are involved in active sensing and regulation of IAI (122–127). For example, LPS simultaneously binds to myeloid differentiation factor 2 and TLR4 that relays signalling of NF-κB through a TLR mediator MyD88, thereby inducing cytokine release (123, 128, 129). Alternatively, TLR4 activation in hAEC can lead to apoptosis *via* Bax/Bcl-2 caspase-3 pathway, thereby triggering a different immune response (122). IL-1R-associated kinase 1 (IRAK1), which is a mediator in TLR/IL-1 pathway, is increased in the hAF in IAI, and showed strong response to intra-amniotic LPS stimulation in animal models (130). IRAK2 plays a similar role and is upregulated in women with preterm IAI (127).

Cytoplasmic PRRs, referred as NLRs, namely NLRP1, NLRP3, NLRC4 and NOD2 were increased at transcriptional level in the presence of IAI in women with preterm labor, although NOD2 was decreased at protein level (118). In a mouse model Faro et al. (132) demonstrated that LPS upregulated NLRP3 inflammasome and downstream caspase-1 and IL-1β at mRNA and protein level, ultimately leading to IAI-induced preterm birth (132). Moreover, several studies in humans have demonstrated increased levels of apoptosis-associated speck-like protein containing a CARD (ASC; adaptor protein involved in inflammasome assembly) in infection and inflammation (133, 220, 239). In addition, functional ASC activation observed by speck formation has been demonstrated *in vitro* in hAEC and hAMSC (240). Assembled and activated NLRP3 inflammasome cleaves pro-caspase-1, which in turn cleaves and activates IL-1β, IL-18 and gasdermin D (134, 135). Gasdermin D induces an inflammatory cell death pyroptosis, which releases cytokines IL-1β and IL-18 into the environment (136, 137). In line with this, several studies have indicated that IAI leads to an increase of caspase-1, gasdermin D and IL-1β and IL-18 in the hAM and hAF (118, 133, 138). The activation of PRR inflammatory pathways has multiple consequences, 1) it can cause cell death *via* various pathways, thereby damaging the integrity of the hAM; 2) PRR downstream signalling changes the expression profile of cells, synthesizing or activating the effector molecules; 3) living cells and cells undergoing inflammatory cell death release the cytokines in the environment, promoting inflammation. Altogether, the PRR activation has evolved to respond to the infection, but in the case of IAI, the inflammation itself is often equally damaging, as it can lead to preterm birth.

### Cytokines

Numerous studies demonstrated an increase in pro-inflammatory IL-1β, IL18, TNFα, granulocyte-macrophage colony-stimulating factor, macrophage migration inhibitory factor, high-mobility group protein 1 (118, 121, 128, 129, 141–146, 201), anti-inflammatory IL10, nicotinamide phosphoribosyltransferase (128, 147, 201, 241),, and regulatory IL-6 (128, 129, 144, 146–151, 201, 241) cytokines in the hAF with IAI or preterm labor conditions. In fact, the IL-6 is considered as an important biomarker of IAI (241–243) and an increased presence of IL-6 (>2.6 ng/ml) in the hAF is used to define inflammation (244). Moreover, chemokines IL-16, IL-8, IP-10, monocyte chemoattractant protein-1, chemokine (C-X-C motif) ligands (CXCLs)-1, -3, -4, -5, -6, -11, and L-selectin have also been upregulated with IAI and related *in vitro* models (128, 146, 147, 154–159). Two comprehensive studies by Bhatti et al. (152) and Romero et al. (140) further reveal the plethora of IAI-induced cytokines in the hAF (140, 152). Of note, the type (e.g. Gram positive, Gram negative, or other) and amount of bacterial pathogens affect the quantity and diversity of cytokine upsurge in the hAF or hAM cells (124, 159, 245, 246). For example, the treatment of amnio-chorionic membrane with heat-inactivated *Escherichia coli* and *Gardnerella vaginalis* strongly induced secretion of IL-1β, while there were no statistically significant changes in IL-1β secretion when the amnio-chorionic membrane were treated with Group B streptococci, *Mycoplasma hominis*, *Ureaplasma urealyticum* or *Ureaplasma parvum*. Similarly, there was a strong increase in TNF-α secretion when the amnio-chorionic membrane were treated with *E. coli*, *Porphyromonas gingivalis* and *G. vaginalis*, while the treatment with *M. hominis*, *U. urealyticum* and *U. parvum* only moderately increased the secretion of TNF-α and there was no change in TNF-α secretion in case of treatment with Group B streptococci (245). In another study the amnio-chorionic membrane were treated with heat-inactivated *E. coli*, *G. vaginalis*, Group B streptococci and *U. parvum*, which led to the increase in IL-6 and IL-10 secretion. On the other hand, there was a statistically significant increase in IFNγ secretion only when the amnio-chorionic membrane were treated with *E. coli* (247). These studies underline the variety of the immune response depending on the bacterial species. Moreover, this together with technical reasons in study design (small sample size, different measurement techniques), as well as natural endogenous variability of the immune response explains why some cytokines were not always consistently increased upon IAI (120, 144, 248).

### Antimicrobial Peptides

Antimicrobial peptides (AMPs) are typically small proteins with a positive charge that directs them to negatively charged bacterial surface. Most commonly they have a broad-spectrum activities, can work synergistically and can disrupt bacterial membrane, causing cell lysis (AMPs are nicely reviewed in (160, 161). AMPs are expressed by the hAM cells (162–165) and are present in the hAF (23, 162, 166–173). They play an important role in the amniotic defence against pathogens and can be constitutively expressed or upregulated in IAI. Most notable are human beta defensins (HBDs)1-3, elafin and secretory leukocyte protease inhibitor (SLPI), which are expressed by hAM cells (162, 174, 175). Specifically, HBD3 was shown to be upregulated in LPS and peptidoglycan presence, both of which are components of bacterial cell wall (176). The amounts of HBD1-3, elafin and SLPI are also increased in the case of IAI (23, 102, 162, 172, 173), though there is some evidence that SLPI and elafin are decreased in preterm PROM (174, 249). Human neutrophil (or alpha) defensins (HNPs)1-3, lysozyme and lactoferrin were also found in the hAF in women with non-complicated pregnancies (170, 182). Moreover, HNP1-3, calgranulin/calprotectin, bactericidal permeability-increasing protein, lactoferrin, lipocalin 2 and cathelicidin family members are increased in the hAF of IAI or preterm labor (100, 101, 150, 152, 166, 168, 169, 171, 177, 178, 183, 179, 180) Overall, the presence and activation of numerous variable AMPs in normal pregnancy and in IAI imply an important role of AMPs in prevention and resolution of infections.

### Lipids and Their Derivatives

Bioactive lipids, such as prostaglandins, are crucial in the onset of labor, specifically in the induction of uterine contractions, and are in fact used to artificially stimulate labor (250–252). Given that they are also involved in inflammation, they might be involved with induction of preterm labor in the case of IAI (184). Even though there are a few conflicting results, most studies show that hAF of IAI patients has increased prostaglandin-prostamide ratio due to an increase of prostaglandins PGE2, PGF2 and PGF2α and decrease of their respective prostamides (155, 185–194). Similar conclusions were obtained in *in vitro* models, in which LPS, IL-1β, TNFα, or whole bacteria stimulation was used to mimic infection and inflammation, though 13,14-dihydro-15-keto-PGF2α levels were decreased in one study (139, 195, 196). Additionally, Gillaux et al. (122) demonstrated an upregulation of prostaglandin-endoperoxide synthase 2, a key enzyme in the synthesis of prostaglandins, through TLR5 and TLR6/2 stimulation in hAECs (122). Interestingly, Maddipati et al. (191) detected a decrease in lipids with anti-inflammatory/proresolution properties in the case of IAI (191). Perhaps most striking is an observation that women with a PGF2α concentration in the hAF above the 95^th^ percentile had a higher rate of IAI, shorter amniocentesis-to-delivery time, and significantly more preterm deliveries (190, 253). Additionally, proinflammatory lipid mediators belonging to the 5-lipoxygenase pathway (5-hydroxyeicosatetraenoic acid and leukotriene B4) are increased in IAI in comparison to sterile inflammation (254).

### Complement System

The complement system is composed of over 40 proteins (serine proteases, receptors and regulators), and it becomes activated through one of the three pathways depending on the trigger – classical, alternative or lectin pathway. After the initial trigger molecule binding, the convertase enzymes cleave proteins C3 and C5, leading to the assembly of membrane attack complex on the microbe membrane that causes the lysis of the pathogen (255, 256). Besides direct destruction of pathogens, the complement system is also a mediator for several innate and adaptive inflammatory processes (256, 257). Hammerschmidt et al. (258) demonstrated that hAF is capable of activating complement, with higher activation in hAF from women with distressed pregnancies, meaning that the women suffered from amnionitis, nonimmune hydrops fetalis, class T diabetes, or severe preeclampsia (258). Another study showed that elevated hAF levels of C3a, but not C5a, were associated with IAI and preterm delivery in women with cervical insufficiency or a short cervix (199). Moreover, the concentration of catalytical subunit of complement factor B (Bb) in hAF was higher in the case of IAI among women with preterm labor or preterm PROM, indicating the activation by alternative complement pathway (200). In addition, regulation of complement activation could be mediated by a membrane-bound complement inhibitor CD59, which was shown to be expressed by hAEC and hAMSC (259). The existing studies show the involvement of complement system in IAI, however, more research is needed to determine the extent of activation, triggered pathways, and the significance of complement activation in this setting.

## A Holistic View on the Interplay Between a Pathogen and the Immune Response in the hAM and hAF

### All Parts of the Innate Immune Response Are Active During IAI

Numerous studies that were mentioned in this review have proven that all parts of the innate immune response are active in the hAM and hAF in the presence of IAI. The components of this response are summed up in **Table 1**, along with their respective function and response to IAI. Other pathways and molecules with less-obvious connection to the immune system are also listed in **Table 1** and their importance as biomarkers is discussed in Chapter 4.3. Given the complexity of the immune response and the consequences, it has become clear that even though the role of the immune response is to prevent or eradicate the pathogens, severe inflammation itself might cause additional undesired complications for the fetus.

### Environmental and Endogenous Factors Influence the Immune Response in IAI

Due to its structural and physiological differences, pathogen species is an important factor in IAI immune response (124, 125, 159, 202, 245, 246). Moreover, there are several environmental and endogenous factors that influence the immune response, not only its strength, but also which of its pathways are triggered. For example, it was observed decades ago that black and African American women have a higher prevalence of poor perinatal outcomes, and later studies show significant differences in hAF immune system response between different races (245, 260–264). For instance, it has been shown that in comparison to African Americans, Caucasians have higher hAF levels of soluble TNF receptor in response to IAI, meaning that TNF-α activity in African Americans is more pronounced (265). Recent advances have shown that these differences are partly a result of genetic predispositions, and multiple single nucleotide polymorphisms were linked to an increased risk for IAI and spontaneous preterm birth (262, 263, 266–269). Nevertheless, environmental factors, such as psychosocial and socioeconomic factors, maternal stress, and epigenetic changes, contribute to the disparities between demographic groups (270–274). For example, psychological stress and depression likely promote preterm birth through inflammation, as is evidenced by increased proinflammatory cytokines IL-6 and TNF-α in the maternal serum (275, 276). Additionally, lifestyle determinants, in particular inadequate diet and nutrition, higher body mass index or obesity, smoking and alcohol consumption, were shown to be relevant risk factors for preterm birth (277–283). For instance, obese patients (class II and III) had increased hAF concentrations of IL-1β and IL-6, and MMP-1, MMP-6, and MMP-13 (284). Finally, the maternal infection with SARS-Cov-2 has been associated with higher incidence of premature delivery, PROM, and neonatal intensive care unit admissions, and the effect of SARS-Cov-2 on pregnancy is currently under intense investigation (54, 55, 285).

### The Potential, Feasibility, and Problems of the Existing IAI Biomarkers

In this review we have focused on the innate immune system components present in the hAM and hAF. However, other molecules and pathways have been monitored in relation to IAI, preterm labor and preterm birth in order to find specific and reliable biomarkers to evaluate the risk of pregnancy complications. For instance, a study by Hong et al. (178) identified several proteins and pathways with altered protein levels in the hAF, such as vascular endothelial growth factor receptor 1 and Fc fragment of IgG binding protein, and pathways of glycolysis, gluconeogenesis, and iron homeostasis (178). In line with this, glucose concentration in the hAF was lower in case of IAI, while hepcidin – a regulator of iron release – was increased (203, 204). Understandably, neutrophil products myeloperoxidase and neutrophil elastase have also been increased in the presence of microbes in the hAF (102, 174, 205), as have been signaling molecules nitric oxide metabolites (197, 202, 206). To name a few more, neurotrophin 3, brain-derived neurotrophic factor, triggering receptor expressed on myeloid cells 1, resistin, soluble human leukocyte antigen-G, angiopoietin-2, cell-free DNA, and histones H3, H4, H2B have all been increased in hAF of women with IAI (100, 201, 207–211). Currently, the samples for determination of IAI are obtained by amniocentesis, which is an invasive approach, while ideally, biomarkers for pregnancy complications should be measured from harmlessly obtained sources. So far, maternal blood, urine, cervicovaginal fluid, and even saliva have been proposed (286–292). Lamont et al. (293) outlined the potential, feasibility and problems of the existing biomarkers, including their retrieval, and hopefully, increasing endeavors and knowledge will bring reliable and safely obtained biomarkers to the clinic in the near future (293).

It is important to note that other tissues, such as placenta and maternal blood, are also involved in the immune response that occurs in the hAF. For example, maternal and fetal neutrophils are recruited to the hAF during IAI (113). Specifically, decidua acts as an important immune cell reservoir that is heavily involved in the immune response in IAI acting from maternal side, and two recent reviews have covered decidual role in IAI (80, 227). Due to the high complexity and extent of the immune system related to the IAI, we have not covered the contributions of other tissues and adaptive immune response in this paper, but they should be considered as well. Understanding the engagement of other relevant tissues in IAI immune response may also prove beneficial in detecting the IAI.

## Concluding Remarks

In conclusion, the innate immune response of the hAF and hAM is a complex and highly regulated process, signified by the involvement of immune cells, mediators and effectors discussed in this paper. However, because of the complexity, an infection might not always be resolved, or the inflammation might become uncontrolled, both of which present a danger of pregnancy complications that may harm the fetus. Thus, it is vital that we understand the pathogenesis of different microorganisms and the variable involvement of the innate immune system of the hAF and hAM. While most of the studies covered in this article were designed to detect specific parts of the immune response, such as immune cells, cytokines, or AMPs, there is a lack of comprehensive and mechanistic studies. Such studies, covering all parts of the immune response in a specific setting, or studies that would reveal detailed regulation of the immune signalling network depending on the pathogen and endogenous factors, would give us a more holistic view on the interplay between a pathogen and the immune response in the hAM and hAF. This understanding is critical in early detection of IAI, its categorization, and sequential treatment options, which will altogether lower the incidence of pregnancy complications and related risks for the fetus.

## Author Contributions

Conceptualization, TŠ, TŽR, MSE, and MEK. Visualization, TŠ, Writing – original draft preparation, TŠ and TŽR. Writing – review and editing, TŠ, TŽ̌R, MSE, and MEK. All authors contributed to the article andapproved the submitted version.

## Funding

This research was funded by the Slovenian Research Agency (project J7-2594, and research core funding no. P3-0108, P1-0198) and the MRIC UL IP-0510 Infrastructure program.

## Conflict of Interest

The authors declare that the research was conducted in the absence of any commercial or financial relationships that could be construed as a potential conflict of interest.

## Publisher’s Note

All claims expressed in this article are solely those of the authors and do not necessarily represent those of their affiliated organizations, or those of the publisher, the editors and the reviewers. Any product that may be evaluated in this article, or claim that may be made by its manufacturer, is not guaranteed or endorsed by the publisher.
